# An Improved Swarm Optimization for Parameter Estimation and Biological Model Selection

**DOI:** 10.1371/journal.pone.0061258

**Published:** 2013-04-11

**Authors:** Afnizanfaizal Abdullah, Safaai Deris, Mohd Saberi Mohamad, Sohail Anwar

**Affiliations:** 1 Artificial Intelligence and Bioinformatics Group (AIBIG), Faculty of Computing, Universiti Teknologi Malaysia, UTM, Johor, Malaysia; 2 Pennsylvania State University, Altoona, Pennsylvania, United States of America; Wayne State University School of Medicine, United States of America

## Abstract

One of the key aspects of computational systems biology is the investigation on the dynamic biological processes within cells. Computational models are often required to elucidate the mechanisms and principles driving the processes because of the nonlinearity and complexity. The models usually incorporate a set of parameters that signify the physical properties of the actual biological systems. In most cases, these parameters are estimated by fitting the model outputs with the corresponding experimental data. However, this is a challenging task because the available experimental data are frequently noisy and incomplete. In this paper, a new hybrid optimization method is proposed to estimate these parameters from the noisy and incomplete experimental data. The proposed method, called Swarm-based Chemical Reaction Optimization, integrates the evolutionary searching strategy employed by the Chemical Reaction Optimization, into the neighbouring searching strategy of the Firefly Algorithm method. The effectiveness of the method was evaluated using a simulated nonlinear model and two biological models: synthetic transcriptional oscillators, and extracellular protease production models. The results showed that the accuracy and computational speed of the proposed method were better than the existing Differential Evolution, Firefly Algorithm and Chemical Reaction Optimization methods. The reliability of the estimated parameters was statistically validated, which suggests that the model outputs produced by these parameters were valid even when noisy and incomplete experimental data were used. Additionally, Akaike Information Criterion was employed to evaluate the model selection, which highlighted the capability of the proposed method in choosing a plausible model based on the experimental data. In conclusion, this paper presents the effectiveness of the proposed method for parameter estimation and model selection problems using noisy and incomplete experimental data. This study is hoped to provide a new insight in developing more accurate and reliable biological models based on limited and low quality experimental data.

## Introduction

Computational systems biology has become an increasingly important research area in the recent years [1], [2]. This field of research is aimed to gain better understanding of how complex biological process respond as a system within living cells. This is often facilitated by using computational models [1], [3], [4]. These models are commonly constructed based on specific mathematical formulations, such as ordinary differential equations (ODEs), to measure the quantity of certain biochemical compounds within a time unit. The development of these models usually involves two stages: network structure identification and parameter estimation [3], [4], [5]. The network structure identification stage is conducted majorly by modelling experts, in which the structure of the ODEs is mathematically verified [3]. Alternatively, the parameter estimation stage is performed to evaluate if the model parameters can accurately simulate the actual processes obtained from the experimental analyses [3], [4].

In general, biological models are equipped with a set of parameters to signify the physical properties of the systems, such as kinetic constants and reaction rates. These parameters are generally difficult to be identified in high-throughput experiments [3]. Instead, they are rather estimated based on the available experimental data. This is usually performed by calibrating the model outputs with the corresponding experimental data. In most cases, nonlinear optimization methods are utilized to find the optimal parameters that can minimize the difference between the model outputs and the corresponding experimental data. However, this is a challenging task as the models are frequently hampered by the nonlinearity of the biological processes [4], [5]. Hence, parameter estimation is usually considered as a nonlinear multi-modal problem, in which the estimation processes may sometimes lead to several insignificant parameters that are less accurate if only based on the actual biological processes [5]. Furthermore, the available experimental data are often incomplete and regularly exhibit a substantial level of measurement noise [5], [6]. These limitations may cause difficulty in finding plausible parameters that represent the actual biological processes. This is a problem of non-identifiability [7], which apprehends the tasks to uniquely estimate the unknown parameters [8], [9], [10].

Currently, there is an increase of the number of nonlinear optimization methods proposed to estimate the parameters in the biological models [1], [4], [11]. The aim of these methods is to find the optimal parameter set which can produce the model outputs that closely fit into the corresponding experimental data. In general, this problem is formulated as the fitness function, usually based on the nonlinear least squares [12]. Conventionally, derivative-based optimization methods are utilized, including maximum likelihood [13] and gradient decent [14] methods. More currently, a local optimization method, namely Extended Kalman Filter (EFK) [15] method, is employed [16]. Lillacci and Khammash [6], [10] introduced an improved EFK method that incorporates the continuous model outputs and the experimental measurements to estimate the parameters using state space searching approach. Additionally, Zheng and co-workers [17] proposed inequality constraints to improve the estimation by using the EFK method. However, both improved methods commonly require the use of model refinement phases to avoid the searching processes from being trapped into the suboptimal solutions. Furthermore, these methods need to consider the limitations of the EFK method that heavily relies on a good set of initial values for both states and parameters in the models [16].

In contrast, several previous works have presented prospective achievements by using meta-heuristic methods [5]. Rodriguez-Fernandez and co-workers [11] employed Scatter Search Algorithm (SSA) [18] to estimate the parameters in benchmark biological models. The study showed that the recombination searching strategy applied by this method was robust to measurement noise in the experimental data. Similarly, Particle Swarm Optimization (PSO) [19] and Genetic Algorithm (GA) [20] methods were also used to estimate the parameters in biological systems, which showed promising results [21], [22]. More recently, evolutionary-based meta-heuristics methods have received remarkable attentions [1], [3], [23]. Generally, these methods utilize evolutionary operations such as crossover, mutation, and selection operations to exploit the information of the solutions in the population. Tashkova and co-workers [3] suggested that the use of Differential Evolution (DE) [24] method is more practical compared to the existing meta-heuristic methods. However, it was also presented that the method may use a substantial amount of computational cost to obtain the best solution [1], [3]. Despite the capabilities, there is no guarantee that these methods will converge to the global optimum solutions [5].

To overcome these limitations, the hybrid meta-heuristics methods are utilized [2], [3], [25]. Commonly, these methods combine different searching strategies from the distinctive methods. Rodriguez-Fernandez and co-workers [26] introduced a new robust hybrid method based on the Evolutionary Strategy (SRES) [27] method. The proposed method had successfully reduced the computational time while handling the measurement noise effectively. In addition, Chen and Wang [28] introduced a new hybrid method which incorporates the DE method with a geometric mean mutation. The method was evaluated using a cellulose hydrolysis model. The experimental results showed that the method was capable to estimate the initial values of the model parameters, in which later were used for gradient-based optimization approach. We had proposed a new hybrid optimization method based on PSO and DE that showed prospective achievement in dealing incomplete and noisy experimental data [29]. In a more recent work [30], we introduced a new hybrid optimization method based on Firefly Algorithm (FA) method [31] and DE methods. To enhance the efficiency of the computational time of the existing methods, the proposed method was used to discriminate the solutions into two sub-populations based on the current fitness values. The sub-population that contained solutions with plausible fitness was exploited for further improvement using a proposed searching strategy based on the FA and DE methods.

In this paper, a new hybrid meta-heuristic method is proposed. The method, called Swarm-based Chemical Reaction Optimization (S-CRO) method, is developed based on the combination of the FA method and a recently proposed evolutionary method, Chemical Reaction Optimization (CRO) [32]. In particular, the proposed S-CRO method is distinguished from the previously proposed method in [29], as the proposed method employs the evolutionary operations of the CRO method to enhance the swarm-based search strategy applied in the FA method, instead of using evolutionary operations of DE method to enhance the PSO method. Thus, this provides a new approach to retain the robustness over the measurement noise that exhibits the experimental data during the searching process [1], [3], [21]. The effectiveness of the proposed method in estimating parameters was evaluated using a simulated nonlinear model [33] and two biological models: synthetic transcriptional oscillators [34], and extracellular protease production [35] models. The performances of the proposed S-CRO method, in terms of convergence to better fitness values and the computational cost used, were compared with those produced by using the standard DE, FA, and CRO methods. In addition, the model outputs generated by the estimated parameters were validated using statistical analysis to address the effectiveness of the method in term of non-identifiability [30]. Furthermore, the method was also validated for model selection, which was performed using the Akaike Information Criterion (AIC) [30], [36]. The paper is organized as follows: Firstly, the problem formulation is introduced and the details of the FA, CRO, and the proposed S-CRO methods are described. The validation analyses for non-identifiability and model selection are also explained. Then, the simulation results are presented. Next, the discussion on the obtained results is addressed, which deliberates the contributions of this work. Lastly, the paper is summarized in the conclusion section.

## Methods

### Problem Formulation

**Figure 1:**
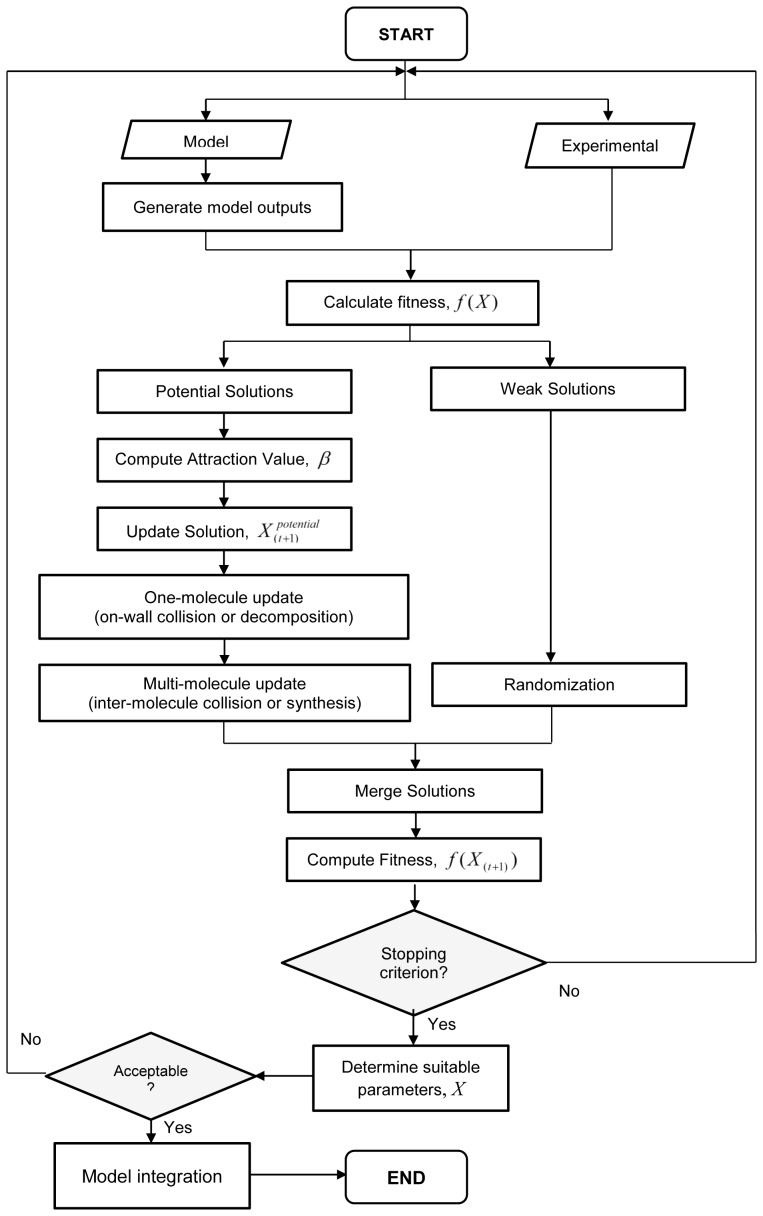
Parameter estimation using optimization method. The parameter estimation procedure begins with a prediction from the model and data obtained from experiments. The model predictions are generated from an ODE solver.

The parameter estimation of the biological models can be formulated as follows. Suppose a model contains a biochemical compound, *s*, that is formed as 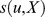, which consists of a set of parameters, 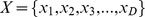, where *D* is the total number of parameters, and the input signal, *u*. Thus, the reaction rate of the compound *s* is given as follows
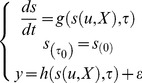
(1)


where *g* and *h* are the nonlinear functions, *τ* is the sampling time, *y* is the model output and *ε* is the measurement noise, which is generated by random Gaussian noise with zero mean [6], [10], [30]. Thus, the parameter estimation problem is aimed to find the optimal parameter set, 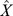, which minimizes the difference between the model output, *y*, and the corresponding experimental data, 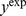. This is commonly performed by using the nonlinear least squared error function, 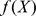, defined as follows:
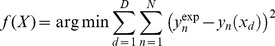
(2)


where *N* is the total number of samples [30]. This function is considered as the fitness function in most optimization methods. Since the experimental data is hampered by the measurement noise and is often incomplete, finding the plausible parameters that may minimize this equation is difficult. Figure 1 shows the general framework of solving parameter estimation problem using nonlinear optimization methods.

### Firefly Algorithm

The FA method is a swarm-based meta-heuristics method [31]. The method is inspired by the natural social behaviours of a firefly population. In nature, the fireflies produce flashing light, which is generated by bioluminescence chemical reactions. The light is used to attract mating partners. The fireflies also use the light as a communication medium to prevent potential preys. In the FA method, the solutions are formulated as the fireflies which carry a vector of variables used to compute the fitness functions. The vector of *i*th solution, 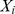, is formed as follows:
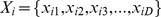
(3)


where *D* is the dimension size of the problem. Each *i*th solution computes the individual fitness value, calculated by a specific fitness function, such as non-linear least squared errors. The fitness value can be represented as the light intensity of the natural firefly. The fitness value of the current *i*th solution is compared with the *j*th neighbouring solutions. If the fitness value of the neighbouring solution is better than the current solution, the distance, 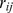, is computed using the standard distance function, such as *Euclidean* distance, as follows [31]:
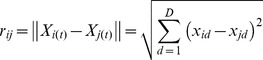
(4)


Using this information, the attractive value of each *i*th solution is further calculated using the following equation [31]

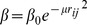
(5)


where for each *i*th firefly with its corresponding *j*th neighbor, 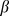 is the attractive value, *μ* is the predefined light absorption coefficient and 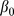 is the initial attractive value [31]. Then, this attractive value is used to update the vector of the *i*th solution:
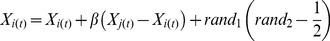
(6)


where 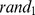 and 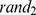 are uniformly distributed random values between 0 to 1 [31]. Thus, this permits the population to move towards the solution that represents the current best fitness value and exploits the searching space more effectively [31]. The searching process is repeated until the maximum number of iterations is reached.

### Artificial Chemical Reaction Optimization

The Chemical Reaction Optimization (CRO) is another meta-heuristic method, which is based on the chemical reactions of molecules to reach low energy stable state [32]. The method manipulates the reactions involving molecules including collision, synthesis and diffusion. In these reactions, the energy is transferred to a stable state is reached. In this method, these molecules are formulated as solutions. Each solution holds two properties: potential and kinetic energies [32]. The potential energy represents the fitness value calculated using the fitness function. On the other hand, the kinetic energy, 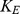, represents a tolerance measurement for the solution to be transformed into a less favourable solution, thus permitting the method to escape the local optima more effectively [32].

In this method, the searching process can be divided into two major actions: single and multi-molecule reactions. The single-molecule reaction usually involves only one solution to be improved using on-wall collision or decomposition processes [32]. Biologically, the on-wall collision occurs when a molecule bumps into a cell wall and then bounces into another direction within the cell. The offspring solutions are mostly less distinctive compared to the parent solution before the collision [32]. For the *i*th solution, the solution intends to gain better fitness from the neighbouring *j*th solution. The vectors in this solution are updated only if the following rule is met
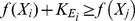
(7)in which the following equation is formed
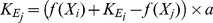
(8)


where 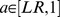, in which *LR* is the loss rate that limits the maximum percentage of kinetic energy lost [32]. Alternatively, the decomposition process occurs when a molecule is diffused into two or more molecules after the collision with the cell wall. The resultant molecules are supposedly to be much different compared to the original molecule. This process is executed if the following rule is met:
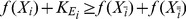
(9)


where 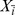 and 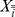 are the offspring solutions from the original solution, 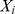, produced after the collision. Based on this rule, a new variable is assigned as follows:
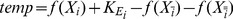
(10)


which is used to generate new kinetic energies, 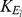 and 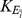, as follow:
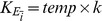
(11)

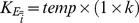
(12)


where *k* is a uniform random number between 0 and 1 [32]. The value is used to generate two newly formed solutions and which are then added into the population.

For multi-molecule reaction, there are two important processes, namely inter-molecule collision and synthesis. The inter-molecule collision involves two solutions that collide with each other and bounce away in two separate directions. The effect of the energy change of the solutions is similar to those in the on-wall collision, except that this process involves two solutions instead of a single solution. The process is performed if the following rule is met [32]:
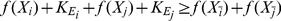
(13)


in which the following variable is produced
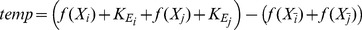
(14)


to generate new kinetic energies, 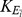 and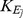, as follow
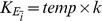
(15)

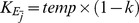
(16)


Thus, these values are used to generate two newly formed solutions and are added into the population. Otherwise, a synthesis process is performed, which involves two solutions to be combined together after the collision. This process is executed if the following rule is accepted:
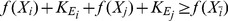
(17)


Based on this rule, the kinetic energy of the newly produced solution, 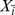, is generated as follows:
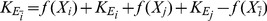
(18)


As a result, the value of 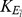 is substantially large compared to 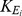 and 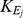 as the value of 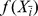
is expected to be equal to 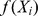 or 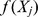 [32]. This process is important to allow the method to escape the local optima more effectively. The process is iterated until the maximum number of iterations is reached.

### Swarm-based Chemical Reaction Optimization (S-CRO)

In this paper, a new hybrid optimization method is proposed based on the CRO and FA methods. The method is developed to introduce the combinatorial searching strategy employed by the evolutionary operations in the CRO method to the swarm-based search strategy of the FA method. This is due to the fact that the evolutionary operations are practical to handle the measurement noise in the experimental data [1], [3], [30]. Basically, the *i*th solution, 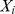, are formulated as follows:
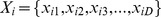
(19)


where 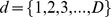 is the number of parameters to be estimated. A number of *NP* solutions are used. The vectors of each solution are initiated randomly within the search space as the following equation:
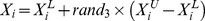
(20)


where 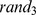
is a uniformly distributed random value between 0 to 1, while 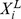 and 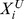 are the predefined lower and upper bound values, respectively. The fitness value of each solution is evaluated. Based on the value, the solution with best fitness value among the population is selected as the current global best solution, 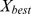.

The S-CRO method incorporates initial selection step, in which the population is sorted based on the fitness values. Then, this sorted population is divided into two major sub- populations. The first sub-population, 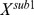, contains a set of solutions that generate potential fitness values whereas the other sub-population, 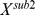, consists of solutions that hold least substantial fitness values [29],[30]. The solutions in the first sub-population are submitted for neighbouring improvement step. In this step, the fitness value of the *i*th solution is compared with its neighbouring solutions. If the value of the *j*th neighbouring solution is better than the *i*th solution, the distance of these solution, 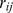, is computed. Then, the attractiveness value, 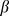, is calculated. According to this value, the vectors of the *i*th solution are updated using equation (6). Next, the *i*th solution is subjected for evolutionary combinatorial step. This is performed by applying the evolutionary operations adopted from the CRO method. Firstly, a random number is generated and if the value is less than 0.5, the *i*th solution is submitted for the on-wall collision (if the value is less or equal to 0.33) or decomposition processes (if the value is greater than 0.33) [32]. Otherwise, if the random value is higher than 0.5, the inter-molecule collision (if the value is greater than 0.7) or synthesis processes (if the value is less or equal to 0.7) are executed into the solution [32]. Since these processes involve two solutions, the *i*th solution and another randomly chosen neighbouring solution are used.

**Figure 2:**
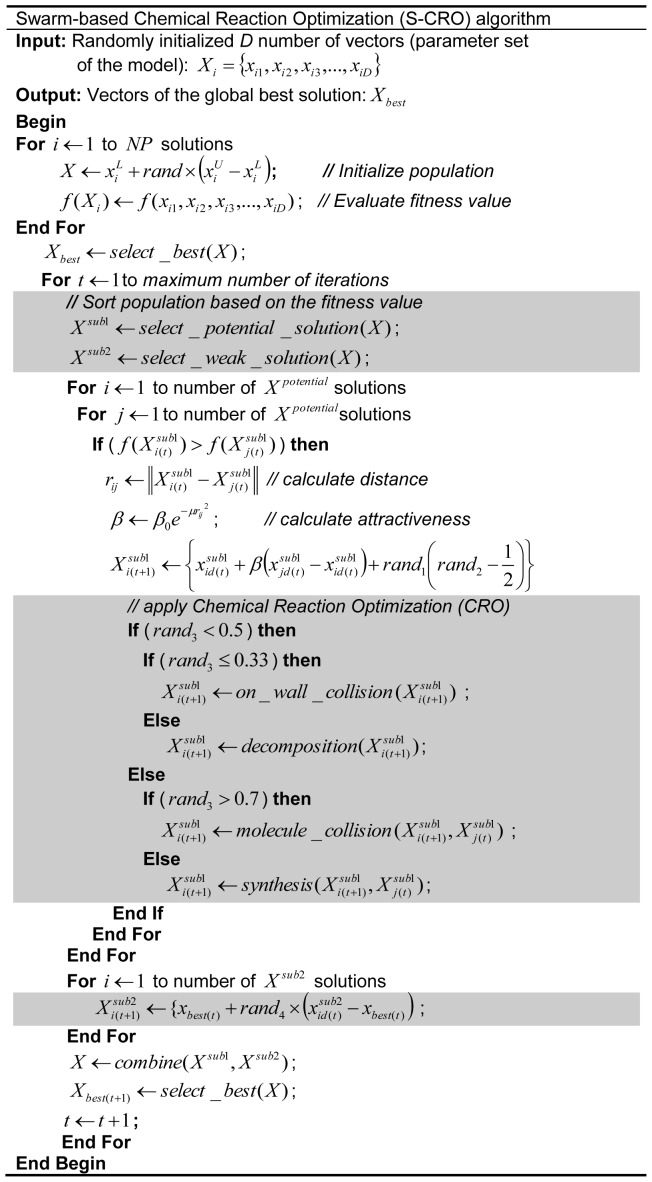
Swarm-based Chemical Reaction Optimization (S-CRO) Algorithm. The proposed S-CRO method is composed of three main steps as indicated by the shaded sections. The first step sorts the population according to fitness into two groups: potential and weak solution groups. In the second step, the potential solutions are subjected to evolutionary operations. In the third step, a random vector update is performed to the weak solutions in order to allow the method to escape from the suboptimal solutions more effectively.

Conversely, the solutions in the second sub-population, 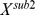, are subjected for random update step. This is performed to ensure that the fitness values of these solutions are improved for the next iterations. Moreover, this step is also implemented to permit the method to escape the local optima more efficiently. The random update is executed using the following equation:
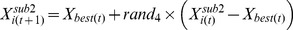
(21)


where 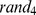 is a random value between 0 and 1 [30]. Different to our work in [29], this step requires vectors of the current best solution to assist the randomization process so that the newly formed weak solutions may consist of potential vectors that will produce better fitness for the next iterations. After this step, the first and second sub-populations are merged to form the updated population. The steps are repeated until the maximum number of iterations is reached. Figure 2 illustrates the algorithm of the proposed S-CRO method.

### Identifiability Analysis

To demonstrate the effectiveness of the proposed method in estimating accurate and reliable parameters, a statistical analysis based on the error variance of the random variables of noise is used [6], [10], [30]. Suppose a model is represented as follows:
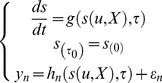
(22)


where 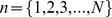 is the number of samples. Thus, the measurement noise is obtained using the following equation:
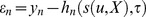
(23)


By executing the methods, an estimated parameter set, 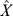, is found. Hence, if 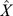 is near to *X*, then 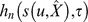 is close to the output 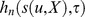, then the variance of 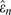 is supposedly close to the variance of 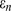. Let 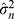 be the variance of 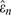. The point estimate of variance 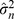 is computed as follows:
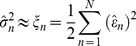
(24)


Subsequently, the interval estimates of 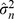 is corresponded to the confident level of 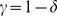 [6], [10], [30], which is formed using the following equation
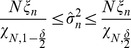
(25)


with a confidence level of 100*γ*%. In other words, if the real variance 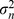 is not lie within these intervals, then the model output *y_n_* could not have been generated by the estimated parameter set, 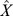. Therefore, the parameter set 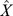 is considered as not plausible for the given experimental data with a confidence level of 100*γ*% [30]. In this paper, a significance level, *δ*, of 0.05 is set, in which giving the confidence level of 95% [6], [10], [30].

### Model Selection

Due to the various uncertainties of the experimental environment, it is important to choose a plausible model that may perform consistent predictions according to the given experimental data [6], [37]. In this paper, the model selection is conducted to assess which environment is more feasible to fit the model prediction. The validation is performed using two approaches. The first approach is suggested by [6] and [10], which is presented in the previous sub-section. Let two distinctive models of the form in Equation (1), which are constructed as follows:
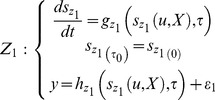
(26)

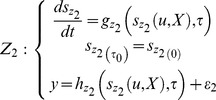
(27)


For both models, the same experimental data are used [6], [10], [30]. Later, the variance points and intervals are computed using these data.

The second approach is applied from [30] and [36], in which the Akaike Information Criterion (AIC) is employed. The AIC validation test is calculated using the following equation:
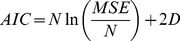
(28)


where *MSE* is the mean squared error that generated from the best fitness value, while *N* and *D* are the number of samples and estimated parameters, respectively [30]. Generally, this equation implies that the model that has smaller AIC value is considered as the best model [36].

## Results

### Simulated Nonlinear Model

**Figure 3:**
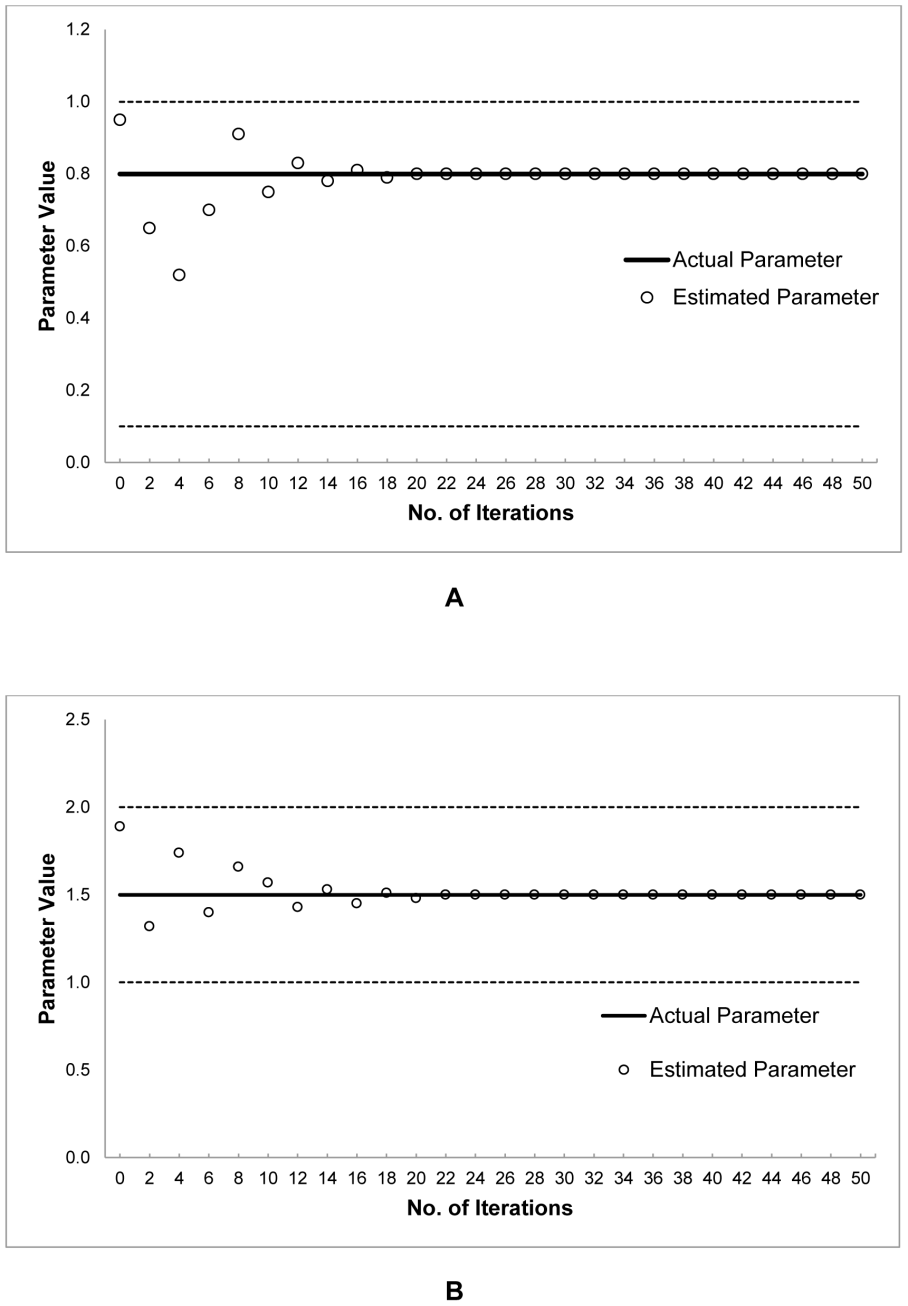
The estimated parameter 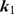 and 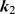 for simulated nonlinear model over the number of iterations by the proposed S-CRO method. The plots show the parameter estimation of the simulated nonlinear model. The dashed lines represent the upper and lower boundaries values, bold lines represent the actual parameter values, and the circles represent the estimated parameter values. Graph A represents the estimated parameter 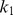 and graph B represents the estimated parameter 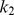.

The proposed S-CRO method was firstly evaluated using the simulated nonlinear model [33]. This was important to show the effectiveness of the proposed method in finding the accurate parameters. The time series data was generated based on the following discrete equations: 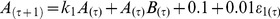
(29)

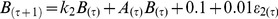
(30)

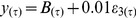
(31)


where the values of parameter 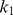 and 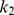 are 0.8 and 1.5, respectively, while 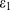, 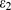, and 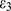 are the independent zero mean noise [16], [33]. The time series data was produced by running the simulation of this model for 800 time points. The upper and lower boundaries of the parameter 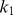 and 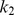
were set as follow: 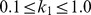 and 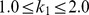. The initial attraction,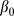, and the light absorption coefficient, *μ*, were fixed to 0.5 and 0.01, respectively. The proposed S-CRO method was executed with 50 iterations. Figure 3 illustrates the results of the estimation by using the S-CRO method. These results showed that the proposed method was capable to accurately estimate the parameters within a relatively small number of iterations.

### Small Scale Model: Synthetic Transcriptional Oscillators

The performance of the proposed method for estimating the parameters in biological models is first evaluated using a synthetic model of transcriptional oscillators [34]. This model is basically a cell-free model, in which the prediction can be studied without the prior knowledge of the existing *in vivo* experiments [34], [38]. The model is proposed to simulate the complex networks of the regulatory perturbation of *deoxyribonucleic acid* (DNA) templates. The model is used to fit in the arbitrary synthetic circuits in a modular fashion [34]. The model is constructed based on the following reactions:
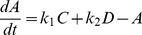
(32)

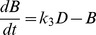
(33)

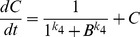
(34)

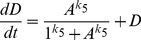
(35)


where *A* and *B* are the ratios of the RNA activator and inhibitor, respectively, meanwhile *C* and *D* are the fractions of ON-state switch Sw21 and switch Sw12, respectively [34]. The values of the parameters 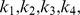
and 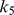, are 0.57 µM, 1.5, 2.5 µM, 6.5, and 6.5, respectively [34]. The model is downloaded from Biomodels database [39].

Table 1 presents the performance comparison of the proposed S-CRO method over the standard DE, FA, and CRO methods in terms of average best fitness values and the efficiency of these methods in utilizing the computational cost. In this paper, the experimental data is generated *in silico*, in which the model predictions are added with 5%, 10%, and 15% of white Gaussian noise [6], [10]. The methods were executed with 100 independent runs in a same workstation powered by Intel Core i5 1.5 GHz of central processing unit (CPU) and 4.0 GB of memory using a 64-bit platform. Each method used 20 solutions and 100 iterations. For the DE method, the mutation and crossover coefficient were set to 2.5 and 1.5, respectively. For the FA and S-CRO method, the initial attraction, 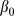, and the light absorption coefficient, *μ*, were fixed to 0.5 and 0.01, respectively. The lower and upper boundaries of the parameters were set as follows: 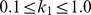, 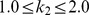, 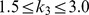, 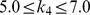, and 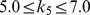.

**Table 1:** Performance comparison of DE, FA, CRO, and S-CRO methods.

	DE	FA	CRO	S-CRO
**Noise: 5%**				
Average Fitness Value	3.92×10^−7^	8.35×10^−5^	8.87×10^−7^	7.21×10^−9^
Standard Deviation	4.35×10^−7^	7.11×10^−5^	6.51×10^−7^	8.43×10^−9^
Computational Time (s)	83.1	103.4	91.9	72.2
**Noise: 10%**				
Average Fitness Value	1.82×10^−3^	5.53×10^−4^	7.01×10^−3^	8.91×10^−6^
Standard Deviation	2.11×10^−3^	4.21×10^−4^	5.25×10^−3^	1.05×10^−6^
Computational Time (s)	98.8	110.8	107.5	81.9
**Noise: 15%**				
Average Fitness Value	2.95×10^−2^	8.22×10^−1^	6.31×10^−2^	5.15×10^−4^
Standard Deviation	2.01×10^−2^	7.42×10^−1^	5.77×10^−2^	1.27×10^−4^
Computational Time (s)	117.8	200.8	135.5	97.9

**Figure 4:**
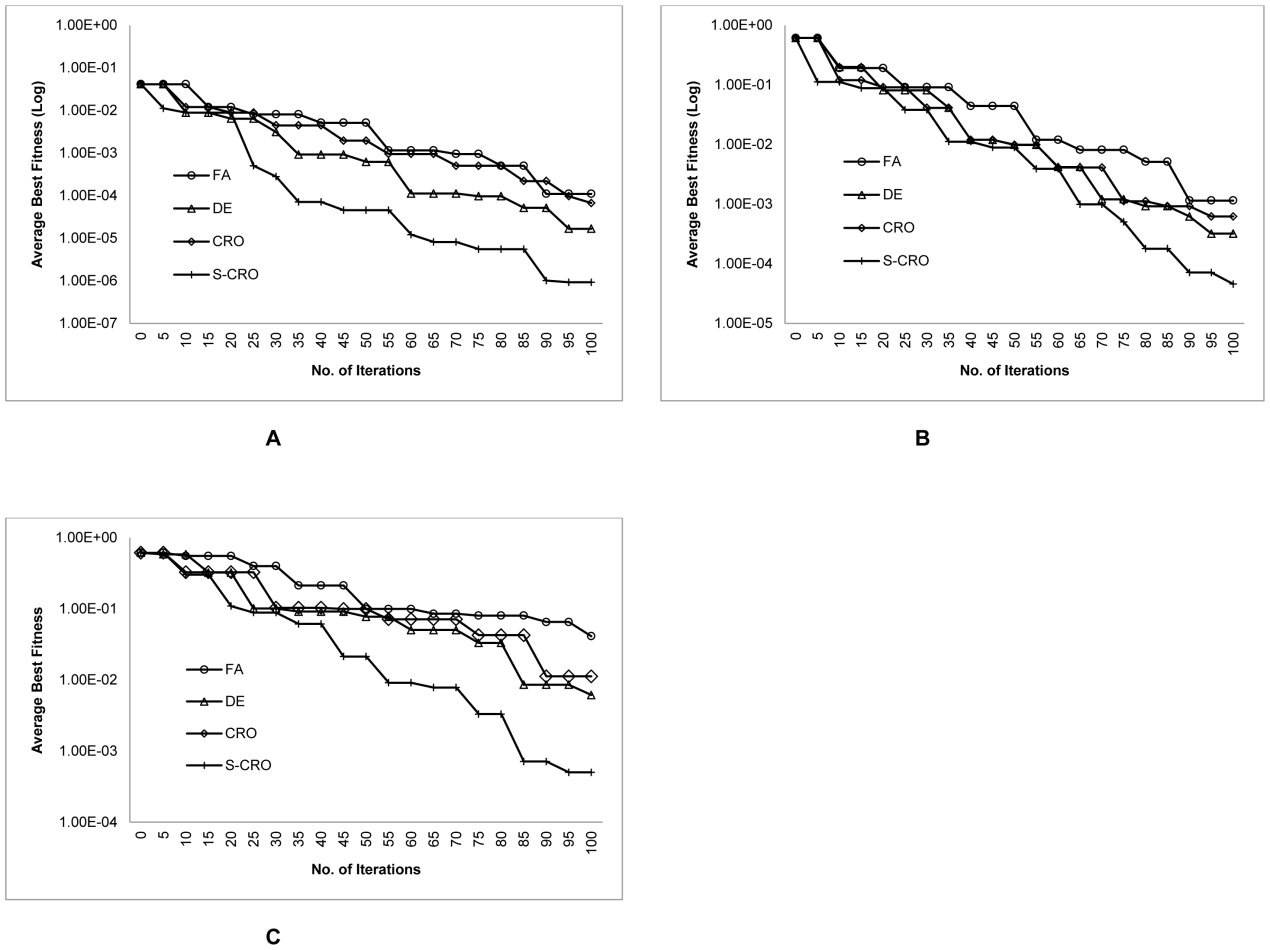
Convergence behaviours of the DE, FA, CRO, and S-CRO methods for the synthetic transcriptional oscillators model. The plots show the average best fitness values of DE, FA, CRO, and the proposed methods in each iteration. Graph A, B, and C represents the convergence behaviours for 5%, 10%, and 15% measurement noise, respectively.

The results exposed that the proposed S-CRO method was capable to find better average fitness values compared to the other methods. In addition, the small number of standard deviation suggested that the fitness values found by the S-CRO method were also consistent for the independent runs. More importantly, the results showed that the proposed method evaluated within an acceptably small amount of computational time. This supports the evidence that the evolutionary operations incorporated with the swarm-based search strategy applied by the S-CRO method could utilize the computational cost more effectively than the other methods. Furthermore, Figure 4 shows the convergence behaviours of the methods in finding the average best fitness values. Initially, the DE and S-CRO methods presented a competitive achievement but as the iterations progressed, the proposed method began showing its advantage due to its capability in converging more frequently. This suggests that the random update step implemented by the S-CRO method was effective to permit the method for escaping the sub-optimal solutions.

**Figure 5:**
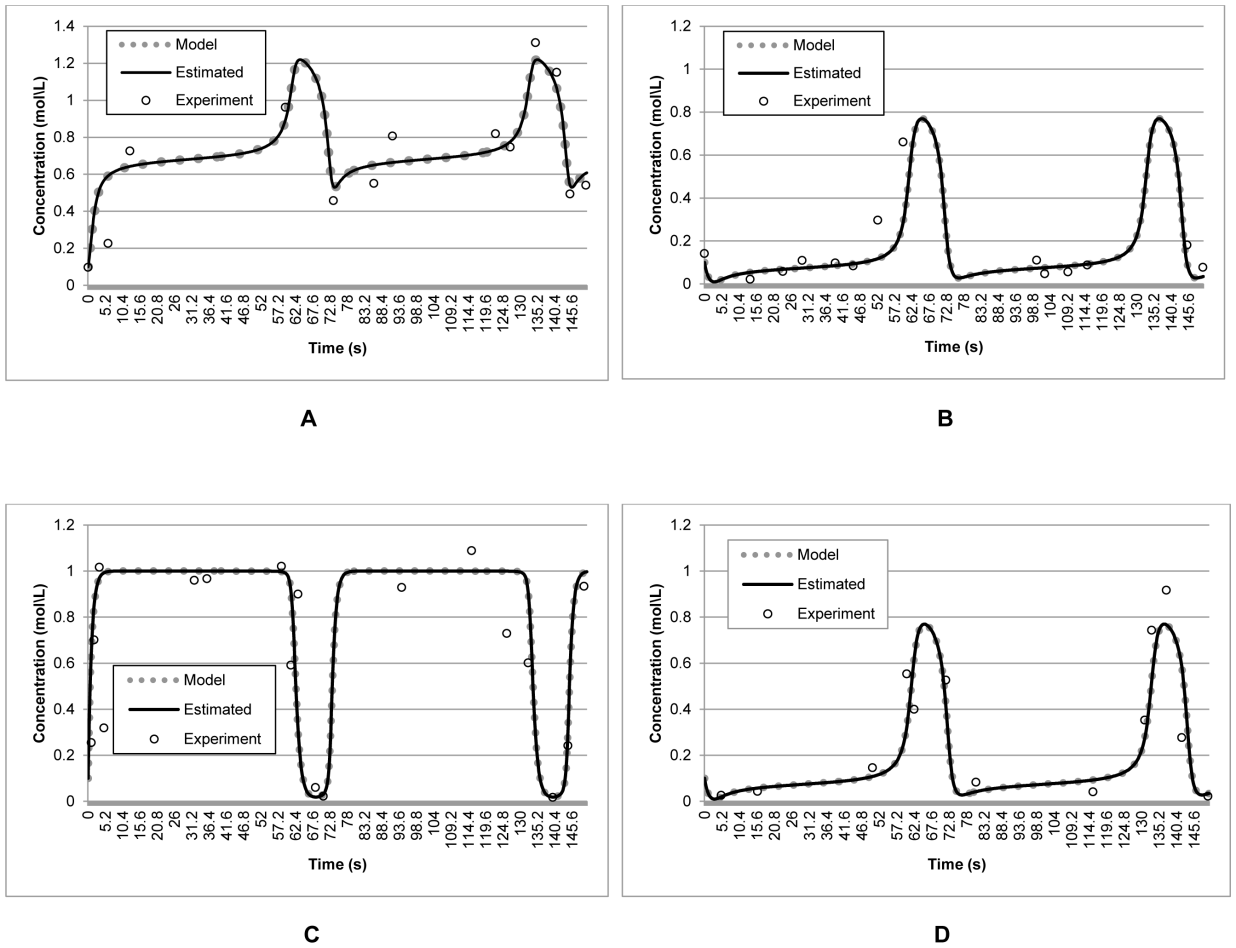
Data fit of model outputs produced by the estimated parameters and the corresponding experimental measurements for the synthetic transcriptional oscillators model. The data points (circles) represent synthetic measurements obtained by adding Gaussian noise to the model prediction (dotted line). The straight lines represent the reconstructed model using the parameters estimated by the proposed S-CRO method. Graph A, B, C, and D represents concentrations of RNA activation, RNA inhibition, ON-state switch Sw21, and ON-state switch Sw12, respectively.

To demonstrate the effectiveness of the method in estimating the plausible parameters using the noisy and incomplete experimental measurements, the model outputs produced by the estimated parameters were compared with those produced by the actual parameters and the experimental measurements. Figure 5 shows that the outputs produced by the reconstructed model were close to those produced by the actual parameters. This shows that the proposed S-CRO method was robust to the noisy and incomplete experimental data. Moreover, Table 2 shows the estimated parameters by the S-CRO method compare to the other methods using noisy and incomplete experimental data. To address the reliability of the S-CRO method, the statistical analysis for non-identifiable parameters is presented. The results are shown in Table 3. According to the analysis, the real variance errors for the RNA activator and inhibitor, as well as ON-state switch Sw21 and Sw12 were 9.28×10^−2^, 8.04×10^−2^, 1.33×10^−1^, and 1.28×10^−2^, respectively. Overall, the variance points computed using the outputs of the reconstructed model were close to the real variance. Prominently, these variance points lay within the variance intervals, which suggest that the model outputs were valid based on the given noisy and incomplete experimental data with 95% confidence level.

**Table 2:** Estimated parameters by DE, FA, CRO, and S-CRO methods using the noisy and incomplete experimental data (15% white Gaussian noise).

Parameter	Actual	DE	FA	CRO	S-CRO
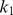 ( µM)	0.57	0.52	1.18	0.52	0.56
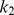	1.5	1.90	1.31	2.09	1.5
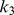 ( µM)	2.5	2.31	3.32	2.45	2.5
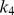	6.5	5.91	6.03	7.21	6.5
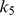	6.5	6.4	5.95	6.4	6.45

**Table 3:** Non-identifiability validation (15% white Gaussian noise).

	RNA activator	RNA inhibitor	ON-state switch Sw21	ON-state switch Sw12
Real Variance ( 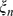 )	9.28×10^−2^	8.04×10^−2^	1.33×10^−1^	1.28×10^−2^
Variance Point ( 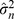 )	9.26×10^−2^	8.04×10^−2^	1.32×10^−1^	1.27×10^−2^
Variance Interval	[8.43×10^−2^, 1.02×10^−1^]	[7.32×10^−2^, 8.89×10^−2^]	[1.21×10^−1^, 1.47×10^−1^]	[1.25×10^−2^, 1.37×10^−2^]
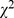 Test	**Pass**			

The S-CRO method was also verified for estimating plausible parameters between two different models. To elucidate this capability, the model defined in Equation 32–35 was modified by changing the values of 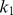, and 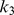 parameters to zero. Hence, the original and modified models were named as 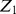 and 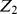, respectively. Table 4 presents the results that compare these models based on the same experimental data. Based on this table, the error variance points computed in the modified model were mostly differed from the real variance points. Moreover, the real variance points did not lie within the calculated variance intervals. Additionally, the validation test showed that the AIC values of the model 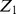 were smaller than the model 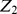. This proved that the proposed S-CRO method was able to discriminate the parameters of these two different models using the same experimental data.

**Table 4:** Model selection validation (15% white Gaussian noise).

Model		RNA activator	RNA inhibitor	ON-state switch Sw21	ON-state switch Sw12
Real Variance ( 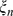 )		9.28×10^−2^	8.04×10^−2^	1.33×10^−1^	1.28×10^−2^
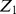	Point ( 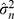 )	9.26×10^−2^	8.04×10^−2^	1.32×10^−1^	1.27×10^−2^
	Interval	[8.43×10^−2^, 1.02×10^−1^]	[7.32×10^−2^, 8.89×10^−2^]	[1.21×10^−1^, 1.47×10^−1^]	[1.25×10^−2^, 1.37×10^−2^]
	AIC	**−2.84×10^4^**			
	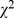 Test	**Pass**			
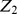	Point ( 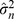 )	6.61×10^−1^	5.11×10^−1^	2.56×10^−1^	9.29×10^−2^
	Interval	[6.01×10^−1^, 7.30×10^−1^]	[4.64×10^−1^, 5.64×10^−1^]	[2.33×10^−1^, 2.83×10^−1^]	[8.45×10^−2^, 1.03×10^−1^]
	AIC	−2.26×10^4^			
	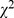 Test	Fail			

### Large Scale Model: Extracellular Protease Production

Naturally, bacterial cells like *Bacillus subtilis* are capable to produce their own nutrient and converge to the steady growth phase by implementing several adaptation strategies. The most substantial strategy used by these bacteria is large scale extracellular protease secretion [35]. Commonly, this process is performed by *subtilisin* (AprE) and *bacillopeptidase* (bpr) genes that encode the involved enzymes to secrete and degrade proteins from the environment. These genes are majorly expressed by DegS–DegU two-component system [35]. The DegS sensor protein is needed to phosphorylate the DegU protein so that the AprE gene expression is triggered [35]. The model is given by the following reactions:
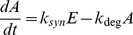
(36)

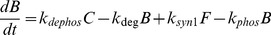
(37)

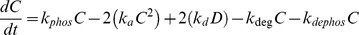
(38)

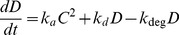
(39)

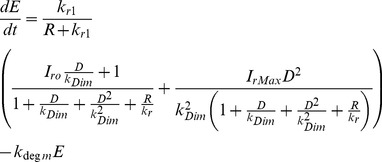
(40)

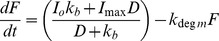
(41)


Where *A*,*B*,*C*,*D*,*E*, and *F* are the concentrations of AprE, DegU, DegUP, Dim, mAprE, and mDegU, respectively [35]. The model contains 17 parameters and the values of these parameters are listed in Table 5. The model is obtained from Biomodels database [39].

**Table 5:** Parameter values of extracellular protease production model.

Parameter	Value	Lower Boundary	Upper Boundary
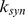 (s^−1^)	0.04	0.02	0.06
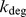 (s^−1^)	0.0004	0.0002	0.0006
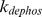 (s^−1^)	0.15	0.05	0.30
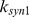 (s^−1^)	0.04	0.02	0.06
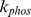 (s^−1^)	0.004	0.002	0.006
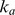 (s^−1^)	0.025	0.010	0.035
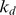 (s^−1^)	0.1	0.05	0.20
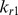 (s^−1^)	7	5	9
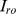 (#/s)	0.02	0.005	0.04
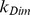 (s^−1^)	12	10	14
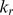 (s^−1^)	7	5	9
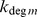 (s^−1^)	0.0099	0.0001	0.0100
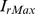 (#/s)	0.4	0.2	0.6
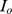	0.004	0.002	0.006
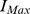 (#/s)	0.048	0.02	0.06
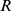 (s^−1^)	7	5	9
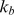 (s^−1^)	7	5	9

A cell volume is assumed to be 10^−15^ litre and the copy numbers (#) is used as a unit for effective concentration. 602 # corresponds to 1 µM/l concentration [28].

**Figure 6:**
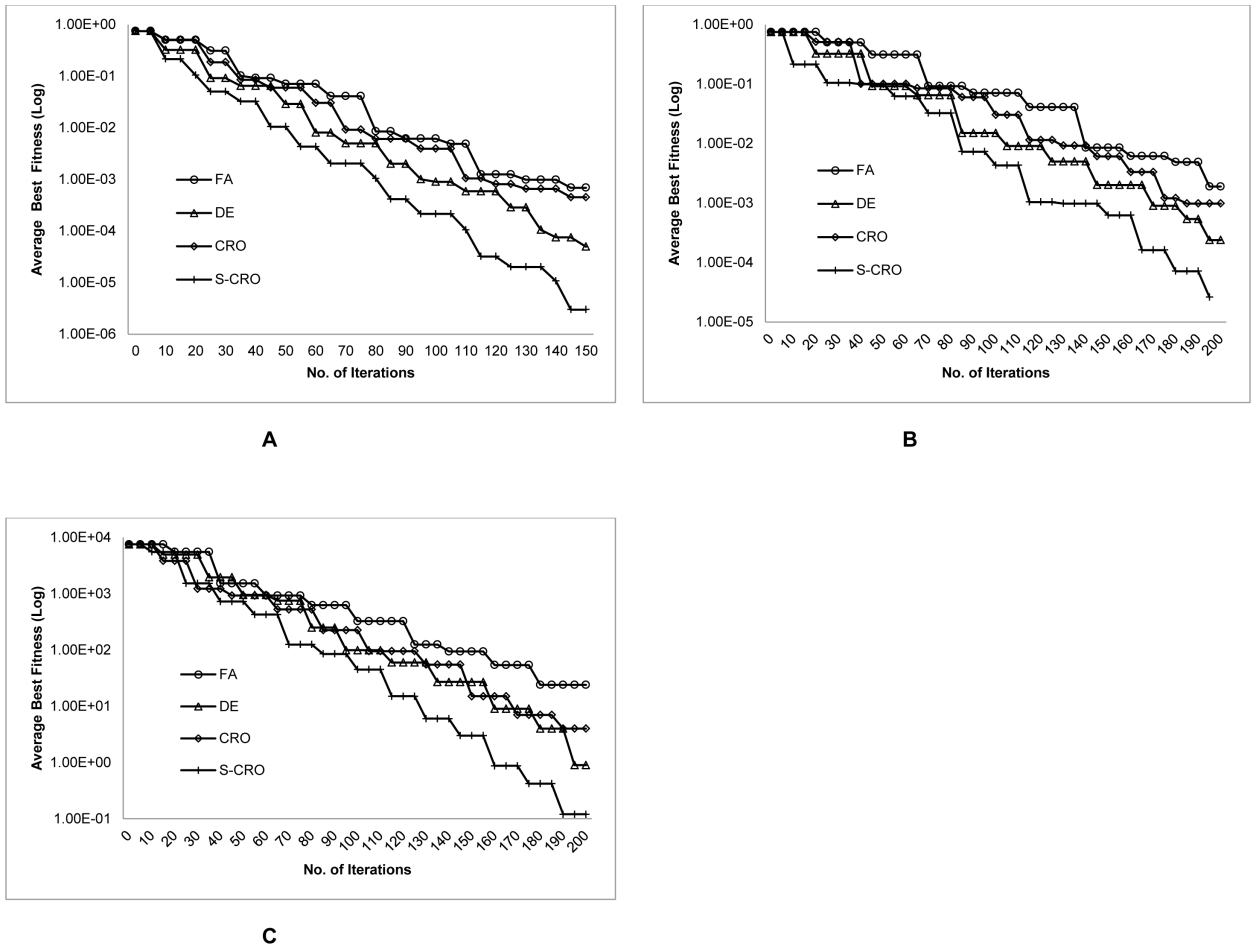
Convergence behaviours of the DE, FA, CRO, and S-CRO methods for the extracellular protease production model. The plots show the average best fitness values of DE, FA, CRO, and the proposed methods in each iteration. Graph A, B, and C represents the convergence behaviours for 5%, 10%, and 15% measurement noise, respectively.

For this simulation, all competitive methods were run using 50 solutions and 200 iterations. For the DE method, the mutation and crossover coefficient were amplified to 3.0 and 2.5, respectively. For the FA and S-CRO methods, the initial attraction and the light absorption coefficient were altered to 0.7 and 0.05, respectively. Table 6 describes the comparison of performance among the DE, FA, CRO, and S-CRO methods. Again, the S-CRO method had shown better average fitness values while maintaining the achievement of finding these values consistently by having the small number of standard deviation. Similar to the previous model, the experimental data for this model is also generated by adding 5%, 10%, and 15% of white Gaussian noise to the model predictions [6], [10]. Based on these results, it is proven that the evolutionary combinatorial step of the proposed method is practical in handling noisy and incomplete experimental data. Similar to the former simulation, the S-CRO method also presented better computational cost utilization compared to the other methods. This can be seen from the relatively small amount of computational time consumed. It was also suggested that the discrimination strategy employed in the initial selection step might have contributed to this result. This was due to the fact that only a specific number of solutions were considered to be evaluated using the method. The convergence behaviours of the involved methods are presented in Figure 6. According to this figure, it is clearly observed that the proposed method converged to the average best fitness values more rapidly than the other methods. Although the performance of the DE method was quite competitive, the random update step of the S-CRO method allowed the method to escape the sub-optimal solution more frequently.

**Table 6:** Performance comparison of DE, FA, CRO, and S-CRO methods.

	DE	FA	CRO	S-CRO
**Noise: 5%**				
Average Fitness Value	1.89×10^−5^	4.14×10^−3^	8.73×10^−5^	7.21×10^−7^
Standard Deviation	2.11×10^−5^	4.99×10^−3^	5.67×10^−5^	1.15×10^−7^
Computational Time (s)	109.3	181.9	120.9	93.4
**Noise: 10%**				
Average Fitness Value	1.67×10^−2^	1.55×10^−1^	9.34×10^−2^	4.77×10^−5^
Standard Deviation	1.82×10^−2^	1.98×10^−1^	8.71×10^−2^	3.99×10^−5^
Computational Time (s)	161.5	256.6	183.9	107.4
**Noise: 15%**				
Average Fitness Value	5.12×10^1^	9.81×10^2^	7.87×10^1^	8.11×10^−2^
Standard Deviation	8.78×10^1^	8.22×10^2^	7.93×10^1^	5.52×10^−2^
Computational Time (s)	243.9	351.3	279.92	150.5

**Figure 7:**
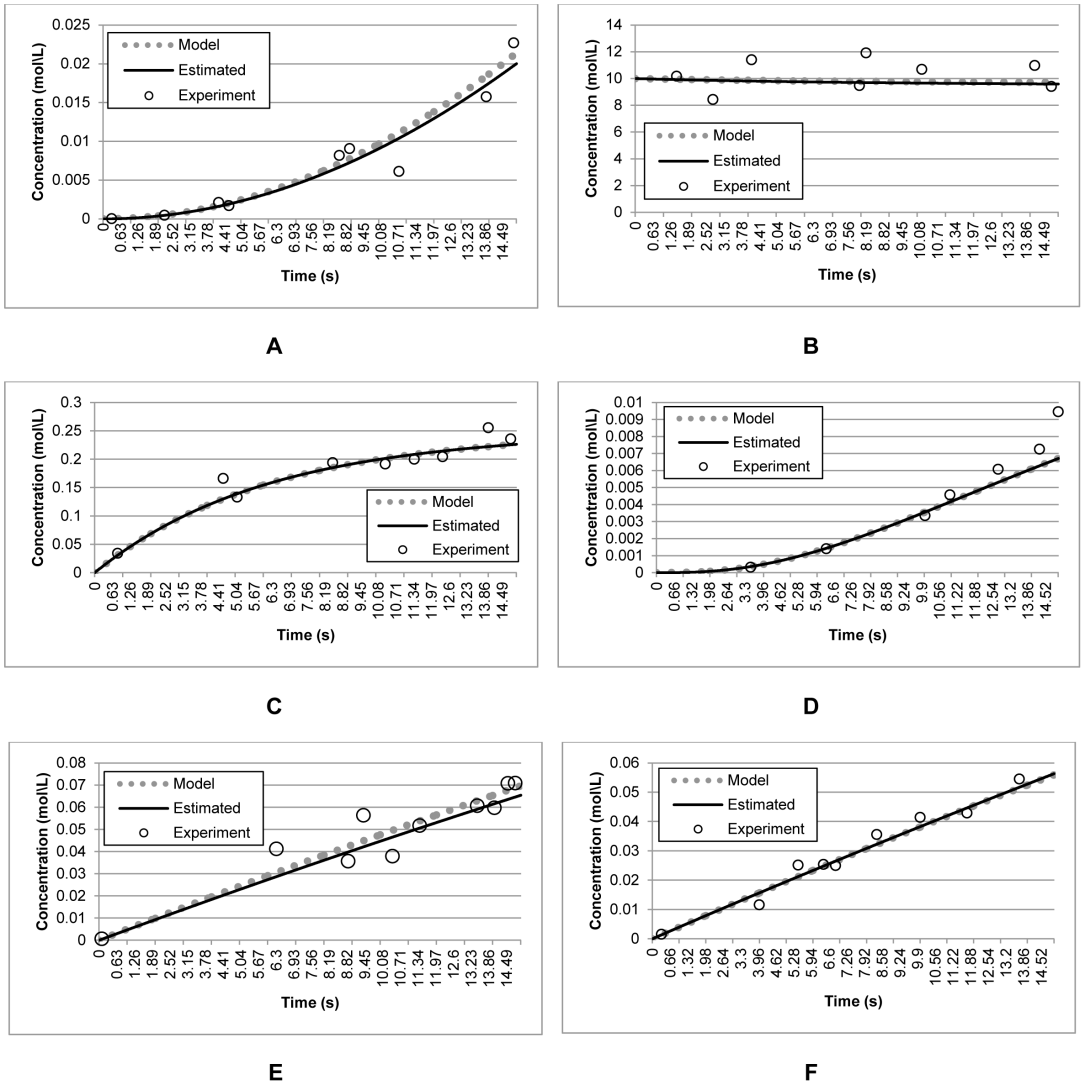
Data fit of model outputs produced by the estimated parameters and the corresponding experimental measurements the extracellular protease production model. The data points (circles) represent synthetic measurements obtained by adding Gaussian noise to the model prediction (dotted line). The straight lines represent the reconstructed model using the parameters estimated by the proposed S-CRO method. Graph A, B, C, D, E, and F represents concentrations of AprE, DegU, DegUP, Dim, mAprE, and mDegU, respectively.

The capability of the proposed S-CRO method in handling the noisy and incomplete experimental measurements is presented in Figure 7. In general, the parameters that had been estimated by the proposed method might have generated the model outputs which closely fitted with those produced by the actual parameters, even though the noisy and incomplete experimental data were used. Table 7 describes the comparison of the estimated parameters by the proposed method over the existing methods. On the other hand, the results of the statistical analysis employed for validating these parameters are described in Table 8. For this model, the real variance error computed were 1.47×10^−5^, 1.59×10^1^, 4.54×10^−3^, 1.83×10^−6^, 3.01×10^−4^, and 1.76×10^−4^ for the concentrations of AprE, DegU, DegUP, Dim, mAprE, and mDegU, respectively. Similar to the results presented in the former experiment, the variance points calculated using the model outputs produced by the estimated parameters were close to the values of the real variance points. Essentially, these variance points lay within the computed variance intervals. This proved that the model outputs produced by these parameters were valid with 95% confidence level. Therefore, the S-CRO method had been considered as robust to the noisy and incomplete experimental data.

**Table 7:** Estimated parameters by DE, FA, CRO, and S-CRO methods using the noisy and incomplete experimental data (15% white Gaussian noise).

Parameter	Value	DE	FA	CRO	S-CRO
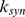 (s^−1^)	0.04	0.047	0.031	0.042	0.04
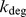 (s^−1^)	0.0004	0.00032	0.00051	0.00049	0.00039
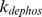 (s^−1^)	0.15	0.22	0.13	0.25	0.149
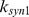 (s^−1^)	0.04	0.051	0.025	0.058	0.04
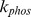 (s^−1^)	0.004	0.0041	0.0052	0.0038	0.004
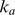 (s^−1^)	0.025	0.0249	0.0245	0.023	0.0249
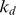 (s^−1^)	0.1	0.099	0.095	0.122	0.099
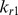 (s^−1^)	7	6.51	7.01	7	7
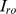 (#/s)	0.02	0.022	0.02	0.015	0.02
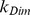 (s^−1^)	12	13.1	12.03	12.8	11.98
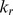 (s^−1^)	7	7.01	5.21	6.95	7
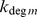 (s^−1^)	0.0099	0.0102	0.0085	0.0092	0.0099
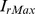 (#/s)	0.4	0.39	0.38	0.39	0.4
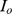	0.004	0.0032	0.0054	0.0036	0.0039
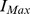 (#/s)	0.048	0.053	0.037	0.052	0.0479
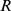 (s^−1^)	7	6.5	6.89	6.5	7
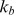 (s^−1^)	7	6.72	7.21	6.8	7

**Table 8:** Non-identifiability validation (15% white Gaussian noise).

	AprE	DegU	DegUP	Dim	mAprE	mDegU
Real Variance ( 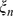 )	1.47×10^−5^	1.59×10^1^	4.54×10^−3^	1.83×10^−6^	3.01×10^−4^	1.76×10^−4^
Variance Point ( 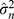 )	1.47×10^−5^	1.59×10^1^	4.55×10^−3^	1.83×10^−6^	3.01×10^−4^	1.76×10^−4^
Variance Interval	[1.38×10^−5^, 1.58×10^−5^]	[1.48×10^1^, 1.70×10^1^]	[4.25×10^−3^, 4.88×10^−3^]	[1.71×10^−6^, 1.96×10^−6^]	[2.81×10^−4^, 3.23×10^−4^]	[1.65×10^−4^, 1.89×10^−4^]
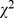 Test	**Pass**					

The results of the model selection are shown in Table 9. In this simulation, the model in Equation 36–41 was adjusted by changing the values of three parameters, 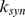, 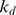, and 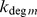 to zero. Similar to the former simulation, the original and modified models were denoted as 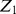 and 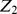, correspondingly. The results presented that the real error variance points of the DegUP, and Dim concentrations did not lie in the computed variance intervals for the modified model. This suggests that the estimated parameters for the modified model might have produced the model outputs that were not valid to the corresponding experimental measurements with 95% confidence level. Moreover, the results also showed that the AIC values of the original model were smaller than the modified model. Again, this simulation showed the effectiveness of the S-CRO method in selecting a plausible model using the given experimental measurements.

**Table 9:** Model selection validation (15% white Gaussian noise).

Model		AprE	DegU	DegUP	Dim	mAprE	mDegU
Real Variance ( 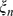 )		1.47×10^−5^	1.59×10^1^	4.54×10^−3^	1.83×10^−6^	3.01×10^−4^	1.76×10^−4^
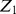	Point ( 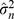 )	1.47×10^−5^	1.59×10^1^	4.55×10^−3^	1.83×10^−6^	3.01×10^−4^	1.76×10^−4^
	Interval	[1.38×10^−5^, 1.58×10^−5^]	[1.48×10^1^, 1.70×10^1^]	[4.25×10^−3^, 4.88×10^−3^]	[1.71×10^−6^, 1.96×10^−6^]	[2.81×10^−4^, 3.23×10^−4^]	[1.65×10^−4^, 1.89×10^−4^]
	AIC	**−3.21×10^4^**					
	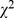 Test	**Pass**					
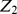	Point ( 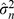 )	1.48×10^−5^	1.59×10^1^	9.59×10^−3^	2.31×10^−5^	3.01×10^−4^	1.79×10^−4^
	Interval	[1.38×10^−5^, 1.58×10^−5^]	[1.48×10^1^, 1.70×10^1^]	[8.97×10^−3^, 9.62×10^−3^]	[2.16×10^−5^, 2.48×10^−5^]	[2.81×10^−4^, 3.23×10^−4^]	[1.67×10^−4^, 1.92×10^−4^]
	AIC	−2.10×10^4^					
	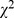 Test	Fail					

## Discussion

Computational systems biology plays an important role in understanding the dynamics of biological systems. This is due to the fact that the biological components involved in the systems often interact with each other to perform specific functions. Therefore, the analyses of individual components are restrictive and impractical [1], [2], [3]. However, this study is commonly hampered by the imperfection of the experimental data obtained in the *in vivo* experimental setups [3], [4], [9]. As a result, the investigations of the complex cellular processes are frequently difficult and ineffective [1], [8]. To elucidate this challenge, a computational modelling approach is exploited. This approach focuses on the design and development of computational models to represent the dynamics behaviours of the biological systems. This is performed by constructing mathematical formulation, namely ODEs, to derive the processes over a specific range of times. These models often depend on a set of parameters that represent the physiological properties of the systems, such as the reaction rates and kinetic constants. These parameters are normally unavailable in the experimental data. Thus, these parameters are rather estimated by fitting the model output with the corresponding experimental data using nonlinear least squares techniques. As the experimental measurements are noisy and incomplete, the estimation of these parameters is usually challenging and often needs the use of practical nonlinear optimization methods [1], [4], [5].

Recent studies have shown a number of optimization methods to estimate the parameters in the biological models. The local optimization methods, especially those that are developed based on the EFK methods, have presented potential achievements in dealing with the experimental measurements [6], [17]. Nevertheless, these methods generally need to be incorporated with the global optimization methods since the EFK methods are only practical to estimate parameters based on the initial values [16]. Due to these limitations, a number of previous works had considered the use of meta-heuristics methods as the methods are generally robust to the measurement noise. Recently, Evolutionary Computation (EC) methods such as GA and DE methods are pondered due to their effectiveness in finding plausible parameters using noisy and incomplete experimental data [1], [5]. Despite of this advantage, the meta-heuristics methods commonly require a significantly huge amount of computational times [1]. This disadvantage often hinders the methods to converge the search for better fitness values frequently. Therefore, hybrid meta-heuristics methods are commonly exploited to overcome these drawbacks [2], [3], [25].

In this paper, a new hybrid optimization method based on the FA and CRO methods is proposed. The new method, called S-CRO method, is developed by incorporating the evolutionary operations adopted from the CRO method to improve the swarm-based search strategy employed by the FA method. The evolutionary operations are often considered practical to handle measurement noise and incompleteness of the experimental data during the estimation of the model parameters [1], [3]. In general, the method is developed to investigate the effectiveness of the new evolutionary strategy, applied using the CRO method into the swarm-based search strategy of the FA method. Thus, this can provide a new approach to handle noisy and incomplete experimental data in the parameter estimation problem. Furthermore, the S-CRO method also introduces a step to rank the population based on the fitness values and divide this population into two sub-populations. This is performed to reduce the computational cost faced by most conventional meta-heuristics methods [3]. The effectiveness of the proposed method, specifically in the parameter estimation problem, was verified by using a simulated nonlinear model, and two biological models: synthetic transcriptional oscillators and extracellular protease production models. The performance of the proposed method was compared with those from the existing DE, FA, and CRO methods. In addition, the proposed S-CRO method was tested for non-identifiability and model selection. These tests were crucial to validate the capability of the proposed method in estimating reliable and identifiable parameters based on the experimental data [6], [9], [17], [30], [36].

The simulation results showed that the proposed method was capable to consistently find better fitness values than the other methods. This provides evidence that the evolutionary operations incorporated with the swarm-based search strategy is practical to handle uncertainty in the experimental data. More importantly, the proposed method also requires an acceptably small amount of computational time. This shows that the initial selection step employed by the method to discriminate the solutions that hold potential fitness values with those that have incompetent fitness values is indeed practical to reduce the computational time. Also, it was observed that the parameters estimated using the proposed S-CRO method could generate model outputs that are valid according to the experimental data. The results showed that the outputs produced by the reconstructed models fitted well with the outputs from the actual parameters even though noisy and incomplete experimental data were used. Different from the work presented in [30], the present method considered the parameter boundaries before the estimation. By doing this, the estimation of the parameters had been improved especially in a model with substantially large number of parameters. In addition, the statistical analysis based on the error variance points and intervals supported that these outputs were produced by the valid parameters estimated by the using proposed method. In terms of model selection, the results presented that the outputs of the modified models had failed the validation test, which suggest that the method is also capable to estimate plausible parameters based on given experimental measurements.

Inclusively, the proposed S-CRO method had shown prospective achievement on estimating parameters. The proposed searching strategy that incorporates the evolutionary operations adopted from the CRO method had presented its effectiveness in handling the measurement noise and incompleteness of the experimental data. Additionally, the initial selection step employed by the proposed S-CRO method had also shown its prominent potential, especially in term of utilizing the computational time. The simulation results suggested that the proposed method is capable of estimating both small and large numbers of parameters. Due to the achievements in the practical non-identifiability, it is preferable to extend the capability of the proposed method in handling structural non-identifiability problem. This is because the problem often involves advance knowledge on the model structure [7], [9], [38], which can lead to further discoveries in selecting feasible routes of the pathways that are particularly important in the commercialized biotechnology engineering.

## Conclusions

In this paper, a new hybrid optimization method is proposed to estimate the parameters of the biological models. The proposed method, S-CRO method, incorporates the evolutionary operations based on the CRO method to enhance the swarm-based searching strategy employed by the FA method. The method is developed to improve the parameter estimation capability of the current optimization methods, especially when noisy and incomplete experimental measurements are involved. The method also utilizes an initial selection step that selects the solutions with feasible fitness values in order to enhance the utilization of computational cost. The effectiveness of the proposed S-CRO method was validated using simulated nonlinear, synthetic transcriptional oscillators, and extracellular protease production models. The simulation results suggested that the proposed method is capable to consistently find better fitness values compared to the other existing methods. Furthermore, the tests also presented that the parameters estimated by using the S-CRO method can produced model outputs that are valid to the corresponding experimental data. Also, the proposed method was tested for non-identifiability and model selection, which showed that the method is capable to estimate reliable parameters and select appropriate models based on the given experimental data.
