# Correction: An Improved Swarm Optimization for Parameter Estimation and Biological Model Selection

**DOI:** 10.1371/annotation/0e890aa4-232d-4fd3-bb82-675dd9fc33ff

**Published:** 2013-05-06

**Authors:** Afnizanfaizal Abdullah, Safaai Deris, Mohd Saberi Mohamad, Sohail Anwar

There were errors in the Funding statement. The correct statement is:

This research was supported by Research University Grant (RUG), project number Q.J130000.2507.04H16, and managed by Research Management Centre (RMC), Universiti Teknologi Malaysia (UTM). The funder had no role in the study design, data collection and analysis, decision to publish, or preparation of the manuscript. 

